# Identification and exploration of potential N6-methylation related mRNAs in endometriosis

**DOI:** 10.3389/fgene.2026.1790963

**Published:** 2026-05-07

**Authors:** Yanan He, Chengcheng Ren, Liang Lei, Jixin Li, Guangmei Zhang

**Affiliations:** 1 Department of Gynecology, The First Affiliated Hospital of Harbin Medical University, Harbin, Heilongjiang, China; 2 Heilongjiang Provincial Hospital, Harbin, Heilongjiang, China; 3 Department of Obstetrics and Gynecology, Shengjing Hospital of China Medical University, Shenyang, Liaoning, China

**Keywords:** endometriosis, m6A-related mRNAs, RAP1A, therapeutic drugs, VCAM1

## Abstract

**Background:**

N6-methyladenosine (m6A) modification regulates the processes of RNA splicing, subcellular localization, translation and stability by changing the RNA structure and the interaction between RNA and RNA-binding proteins to ensure the timely and accurate expression of genes. In this study, we investigated m6A-related mRNAs and for the first time explored effective prevention and treatment targets in endometriosis (EM).

**Methods:**

By arraystar m6A-mRNA epitranscriptomic microarray, biological information analysis technologies, and validation of other databases, aberrant m6A-related mRNAs were uncovered, as well as efficient therapeutic drugs.

**Results:**

*FN1, VCAM1, RAP1A, BRCA1, CCNA2* and *CDK1* might be vital m6A-related mRNAs, and *VCAM1* and *RAP1A* may be the most important two. A few crucial small-molecule agents supply new views for the treatment of EM.

**Conclusion:**

These results demonstrated novel insights into m6A modification of EM and revealed potential biomarkers and precision medicine strategies for EM.

## Introduction

Endometriosis refers to the process of growth and lesions of active endometrial tissue outside the uterine cavity, which is a common disease and refractory disease in women of childbearing age, with an incidence of 10%–15% and a significant upward trend (Giudice and Kao, 2004). Although it is benign lesions, its biological behavior is similar to that of malignant tumors, manifested as adhesion, implantation, infiltration, metastasis and recurrence (Burney and Giudice, 2012). This kind of benign nature and similar malignant behavior has always been a very difficult problem in gynecological clinics (Halme et al., 1984). At present, there are many hypotheses about the pathogenesis of internal heterogeneity, including the theory of menstrual blood reflux, intestinal metaplasia, immune system abnormalities, genetic factors, etc., but none of them can explain the diversity of clinical manifestations and the complexity of treatment, and the source molecular mechanism remains to be clarified (Wang et al., 2020).

In recent years, DNA methylation and protein post-translational modification have laid a good research foundation for epigenetics (Jones et al., 2010). On this basis, RNA methylation modification gradually caused a new wave of epigenetic research (Krishnamoorthy and Decherney, 2017). N6-methyladenosine (m6A) is a dynamic reversible methylation modification that occurs on the sixth nitrogen atom of adenine, widely present in eukaryotic mRNAs, mainly distributed near stop codons, 3′untranslated regions and exon regions, is a common modification of RNA methylation, and is also a chemical modification with the highest RNA abundance in eukaryotic cells (Yang et al., 2018). Similar to DNA methylation, RNA m6A modification is also catalyzed by methyltransferase and demethyltransferase-mediated mediation (Zhao et al., 2020). Methyltransferases include METTL3, METTL14, etc., demethyltransferases include FTO, ALKBH5, etc., and m6A-binding proteins include YTHDF1, YTHDF2, etc (Huan et al., 2021; Lee et al., 2020). Recently, after in-depth research on the regulation between the dynamic balance of methylation and demethylation of m6A modification, it has been found that RNA m6A modification is closely related to RNA metabolism, including the regulation of RNA structure, variable cleavage of RNA, translation and degradation of RNA, and transport of RNA (Li et al., 2021; Jiang et al., 2020; Ries et al., 2019). In addition, RNA m6A modification can also affect the occurrence and development of a variety of biological processes and diseases (Han et al., 2020). For example, METTL3 and METTL14 drive aging-related secretory phenotypes; FTO is associated with obesity; YTHDF1 promotes stress particle formation.

At present, no research has been reported on whether RNA m6A modification is involved in the occurrence and development of internal heterogeneity (Li H. et al., 2022). From the perspective of RNA m6A modification, this study explores the role and potential regulatory mechanism of RNA m6A modification in endochromatics, so as to further deepen the understanding of the occurrence and development of endocytosis.

In this article, data from arraystar m6A-mRNA epitranscriptomic microarray were analyzed and integrated to screen out abnormal m6A-related biomarkers. A protein‒protein interaction (PPI) network was established to identify vital nodes. In addition, the GSE5108 database and Turku Endometriosis Database were used to validate the key genes. Finally, we evaluated drugs for endometriosis and provided new ideas for diagnostic and therapeutic targets.

## Methods

### Collection of tissue samples

A total of 15 ectopic endometriotic samples and 15 normal endometrium were collected. Ectopic endometriotic samples from patients who underwent surgery without chemotherapy and perimenopausal at the First Affiliated Hospitals of Harbin Medical University. Normal samples from patients who underwent hysterectomy because of uterine leiomyoma. All normal endometrium were pathologically confirmed absence of endometriosis and other endometrial disorders. All patients had regular menstrual cycles. Women receiving hormone therapy or with other serious disease were excluded. To minimize confounding effects, all samples were collected at the same menstrual phase, with standardized tissue preservation and uniform laboratory procedures. Potential confounding influences were further controlled by statistical adjustment and principal component analysis (PCA). The research was approved by the ethics committee of the First Affiliated Hospital of Harbin Medical University. All tissues were immediately preserved in liquid nitrogen until use. The diagnosis of each specimen has been revealed based on histopathological evaluation.

### Arraystar m6A-mRNA epitranscriptomic microarray

Ectopic endometrium and normal endometrium tissues for microarray were collected from six patients (3V3). A total of RNA was quantified applying the NanoDrop ND-1000 and RNA integrity was evaluated by Bioanalyzer 2100 or Mops electrophoresis. RNAs were incubated with anti- N6-methyladenosine (m6A) antibody. The m6A-methylated RNAs were eluted from the immunoprecipitated magnetic beads, and unmodified RNAs were achieved from the supernatant. The modified and unmodified RNAs were augmented as cRNAs and labelled with Cy5 and Cy3, respectively, though Arraystar RNA Labeling protocol. The cRNAs were hybridized onto an Arraystar Human mRNA Epitranscriptomic Microarray (8 × 60 K, Arraystar). After washing the slides, the arrays were scanned with an Agilent Scanner G2505C. The raw microarray data are available in GEO (GSE248593), while other data are included in the supplementary materials.

Agilent Feature Extraction software (version 11.0.1.1) was applied to analyze acquired array images. Raw intensities of Cy5-labelled and Cy3-labelled cRNAs were normalized to the average of log2-scaled intensities of the Spike-in RNA. Afterwards,the probe signals with present or marginal quality control flags in at least three out of six samples were retained for next analyses. The quantity of m6A modification was calculated as the m6A methylation level based on the Cy5-labelled normalized intensities. Aberrantly m6A-methylated RNAs between ectopic endometrium and normal endometrium tissues were identified by filtering with the fold change and statistical thresholds. Hierarchical Clustering was used to present the distinguishable m6A-methylation pattern among tissues. Aberrantly m6A-methylated mRNAs were analyzed for GO and KEGG pathway enrichment.

### Protein-protein interaction network analysis and hub gene selection

Differently m6A-methylated mRNAs were uploaded to STRING database to establish their Protein-protein interaction (PPI) network. The minimum required interaction score was set at >0.9 and the disconnected genes were hidden in the network. The Cytoscape software was used to present the PPI network and select hub genes. Venn diagram was used to show the top ten modules in betweenness, degree and closeness three different algorithm analysis through Cytoscape. The topological parameter thresholds were defined as: degree (top 10%), betweenness centrality (top 5%–10%), and closeness centrality (top 5%–10%). To ensure the robustness of hub gene identification, threshold sensitivity analysis was conducted by adjusting the cutoff criteria. The results confirmed that core hub genes including VCAM1, RAP1A and FN1 were consistently detected across different threshold settings.

### Validation of the novel hub genes in other databases

For other database identification, the authors sought the gene expression profile dataset GSE5108, which was derived from the platform GPL2895 (GE Healthcare/Amersham Biosciences CodeLink Human Whole Genome Bioarray) and the Turku Endometriosis Database (https://endometdb.utu.fi), which was used to confirm the hub genes in endometriosis samples. Furthermore, ROC analysis was showed the diagnostic values of the hub genes using GraphPad Prism 9.3.1.

### Discovery of small molecular drugs

The differently m6A-methylated mRNAs were introduced into the CMap database (https://clue.io/), a publicly accessible validation of small molecular drugs website. PubChem (https://pubchem.ncbi.nml.gov) was used for 3D structure of the established potential small molecules. The STITCH database (http://stitch.embl.de) was applied to search the connection between hub genes and small molecules.

### Molecular docking analysis

Firstly, we downloaded the 3D structures of small molecules from the PubChem database; imported the structures into ChemBio3D Ultra 14.0 and set Minimum RMS Gradient to 0.001; and then performed hydrogenation, charge calculation, charge distribution, the rotatable key set in the Autodock Tools-1.5.6. Secondly, we downloaded VCAM1 and RAP1A protein structures from the PDB database; used Pymol2.3.0 to remove protein crystal water, primitive ligands; and then imported protein structures into Autodock Tools. Thirdly, PyMOL2.3.0 was used to analyze the interaction mode of the docking results.

### RNA extraction and quantitative real-time PCR (qRT-PCR)

Ectopic endometrium and normal endometrium tissues for microarray were selected from 24 patients (12V12). This was an exploratory study, and the sample size was limited by specimen availability. Total RNA was extracted by TRIzol reagent (Invitrogen, CA, USA) in ectopic and normal samples. RNA was reverse transcribed into cDNA using EasyScript™ Reverse Transcriptase (Beijing TransGen Biotech, China). RNA expression levels were detected by qPCR using SYBR Green PCR MasterMix (Takara, Japan) and TaqMan MicroRNA Assay Kit on a CFX96 real-time PCR Detection System (Bio-Rad, Hercules, CA, USA). The relative expression of *VCAM1* and *RAP1A* was normalized to GAPDH, and calculated by 2^−ΔΔCT^ method. PCR reaction conditions were as follows: 95 °C for 2 min, followed by 40 cycles of amplification at 95 °C for 15 s and 60 °C for 1 min. Primer sequences used in our research are presented in Table 1.

**TABLE 1:** qRT-PCR primer sequences used in our research.

Primer	Sequence (5′–3′)
VCAM1 forward	GATTCTGTGCCCACAGTAAGGC
VCAM1 reverse	TGGTCACAGAGCCACCTTCTTG
RAP1A forward	ACTTACAGGACCTGAGGGAACAG
RAP1A reverse	CCTGCTCTTTGCCAACTACTCG
GAPDH forward	CAGGAGGCATTGCTGATGAT
GAPDH reverse	GAAGGCTGGGGCTCATTT

### Statistical analysis

Student’s t-test was used to compare the expression of aberrantly m6A-mRNAs. Statistical tests were set as two-sided, and the difference was considered significant at p < 0.05 (ns, p ≥ 0.05; *, p < 0.05; **, p < 0.01; ***, p < 0.001).

## Results

### Determination of aberrantly m6A-methylated mRNAs in endometriosis

**FIGURE 1:**
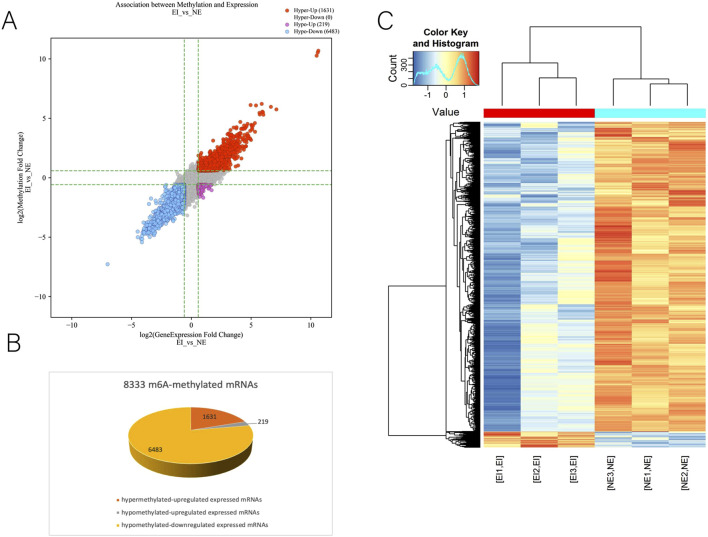
Identification of 8333 m6A-methylated mRNAs in endometriosis. **(A)** Volcano plot of 8333 m6A-methylated mRNAs. Red, purple and blue data points represent hypermethylated upregulation, hypomethylated upregulation and hypomethylated downregulation, respectively. **(B)** Identification of 1631 hypermethylated-upregulated expressed mRNAs, 219 hypomethylated-upregulated expressed mRNAs and 6483 hypomethylated-downregulated expressed mRNAs in endometriosis. **(C)** The heatmap of differential m6A methylation levels in the ectopic and normal endometrium. Each row represents a m6A-methylated mRNAs and each column represents a sample. NE: normal endometrium; EI: Ectopic endometrium.

M6A–mRNA epitranscriptomic microarray was proceeded with the samples from ectopic endometrium and normal endometrium. To contrast two groups for aberrantly m6A methylation level, the fold change (*FC*) and statistical significance of the difference (p-value) were calculated for each transcript. The default thresholds are |*FC*| ≥2 and p-values <0.05. Multiple testing correction was performed using the Benjamini–Hochberg (BH) method to control the false discovery rate (FDR). Raw microarray data were subjected to background correction and quantile normalization. Principal component analysis (PCA) was utilized to evaluate and minimize batch effects, and samples from the same batch were equally distributed between case and control groups. Raw microarray data were processed with background correction and quantile normalization. Principal component analysis (PCA) was applied to assess and minimize batch effects, and samples within each batch were equally allocated across case and control groups. A total of 8333 m6A-methylated mRNAs were obtained, including 1631 hypermethylated-upregulated expressed mRNAs, 219 hypomethylated-upregulated expressed mRNAs and 6483 hypomethylated-downregulated expressed mRNAs (Figures 1A,B). Moreover, the clustering analysis displayed differential m6A methylation levels in the ectopic and normal endometrium (Figure 1C).

### Functional enrichment analysis of hyper- and hypo-methylated m6A methylated mRNAs

**FIGURE 2:**
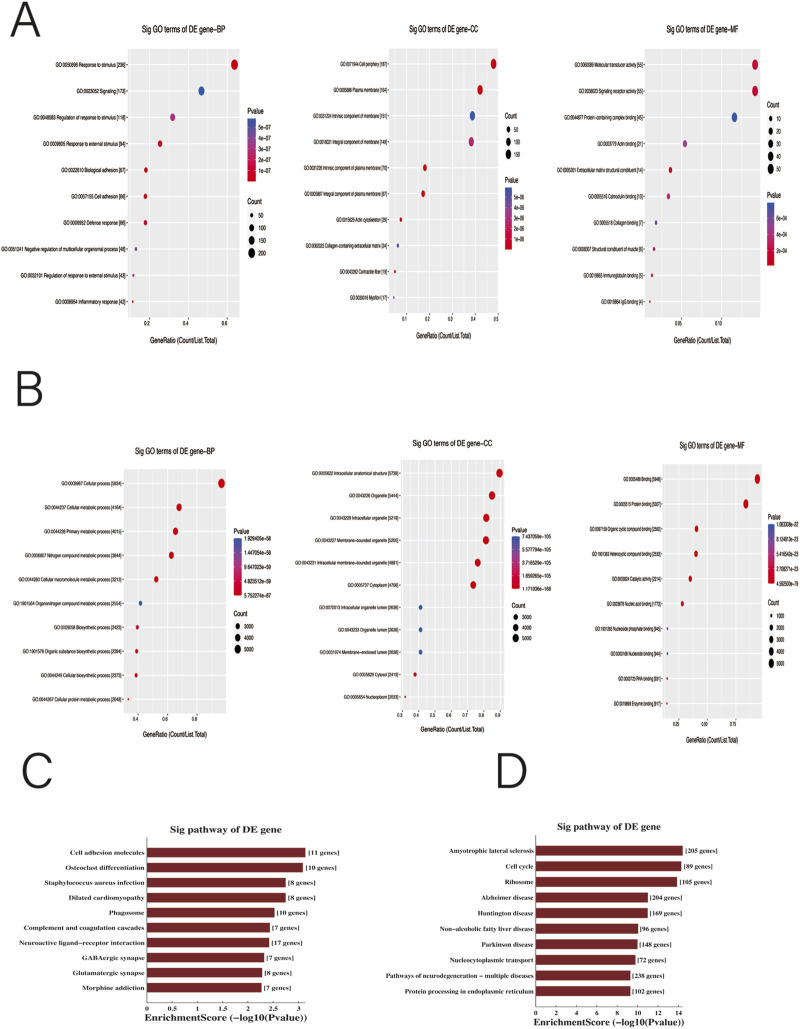
Gene ontology (GO) and Kyoto Encyclopedia of Genes and Genomes pathway analyses (KEGG). **(A,B)** GO analysis of the top 10 hypermethylated m6A mRNAs **(A)** and hypomethylated m6A mRNAs **(B)** in endometriosis. biological processes (BP), cellular components (CC) and molecular function (MF) (p < 0.05). **(C,D)** Kyoto Encyclopedia of Genes and Genomes pathways analysis of 1631 hypermethylated m6A mRNAs **(C)** and 6702 hypomethylated m6A mRNAs **(D)** in endometriosis (p < 0.05).

Gene ontology (GO)and Kyoto Encyclopedia of Genes and Genomes (KEGG) pathway analyses exhibited the biological function of differentially m6A-methylated mRNAs in endometriosis. The top 10 enriched biological processes (BP), cellular components (CC) and molecular function (MF) genes with hyper-and hypo-methylated m6A were verified. In BP analysis, hypermethylated genes were mainly enriched in the terms ‘response to stimulus’, ‘signaling’ and ‘regulation of response to stimulus’. CC analysis revealed enrichment predominantly in the term ‘cell periphery’,‘plasma membrane’ and ‘intrinsic component of membrane’. In addition, MF analysis uncovered enrichment in the terms ‘molecular transducer activity’, ‘signaling receptor activity’, and ‘protein-containing complex binding’ (Figure 2A). Hypomethylated genes were remarkably enriched in the terms ‘cellular process’ in biological process analysis. In CC analysis, the hypomethylated genes were obviously enriched in the terms ‘intracellular anatomical structure’ and ‘organelle’. Additionally, MF analysis indicated enrichment with the terms ‘binding’, and ‘protein binding’ (Figure 2B). The result from KEGG analysis demonstrated that hypermethylated genes were strikingly enriched in the signaling pathway of cell adhesion molecules, osteoclast differentiation and *staphylococcus aureus* infection, while the hypomethylated genes were mainly linked to amyotrophic lateral sclerosis, cell cycle and ribosome (Figures 2C,D). These pathways elucidated that differences in the m6A methylation profiles maybe have crucial effect in the etiopathogenesis of endometriosis.

### PPI networks analysis of the hub genes

**FIGURE 3:**
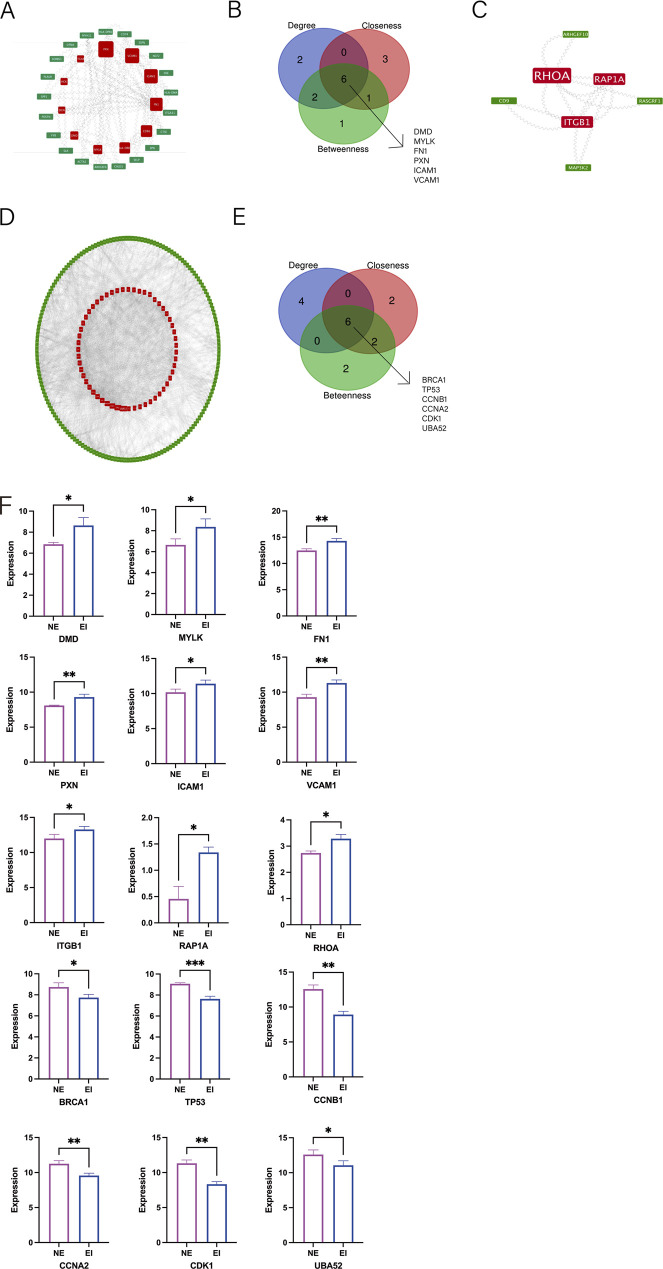
The protein-protein interactionnetwork(PPI), module and identification of hub m6A mRNAs. **(A)** The PPI network of hypermethylated-upregulated m6A mRNAs. **(B)** The Venn picture of the top ten hypermethylated-upregulated m6A mRNAs. **(C)** The PPI network of hypomethylated-upregulated m6A mRNAs. **(D)** The PPI network of hypomethylated-downregulated m6A mRNAs. **(E)** The Venn picture of the top ten hypomethylated-downregulated m6A mRNAs. **(F)** The expression of hub genes in NE and EI tissues (3V3).

The PPI network of hypermethylated-upregulated expressed mRNAs consisted of 40 nodes and 45 edges (Figure 3A). The cytoHubba plug-in was applied to selected the top ten hub m6A-related mRNAs by three different algorithms–degree, betweenness and closeness. The hub mRNAs were *DMD, MYLK, FN1, PXN, ICAM1 and VCAM1* overlapping three different features among the hypermethylated-upregulated expressed mRNAs (Figure 3B), which might have important roles on m6A methylation in endometriosis. The PPI network for hypomethylated-upregulated expressed mRNAs is unraveled in Figure 3C. Three mRNAs *ITGB1, RAP1A* and *RHOA* which can be considered key mRNAs are clearly shown in the network.

The PPI network for hypomethylated-downregulated expressed mRNAs is displayed in Figure 3D. To validate the hub nodes from hypomethylated-downregulated expressed mRNAs, the authors also chose the top ten mRNAs from the three topological features above and harvested that six mRNAs, *BRCA1, TP53, CCNB1, CCNA2, CDK1,* and *UBA52* (Figure 3E). A total of 15 mRNAs were screened from the PPI network for further validation from clinical tissues (Figure 3F).

### Validation of the novel hub mRNAs in other databases

**FIGURE 4:**
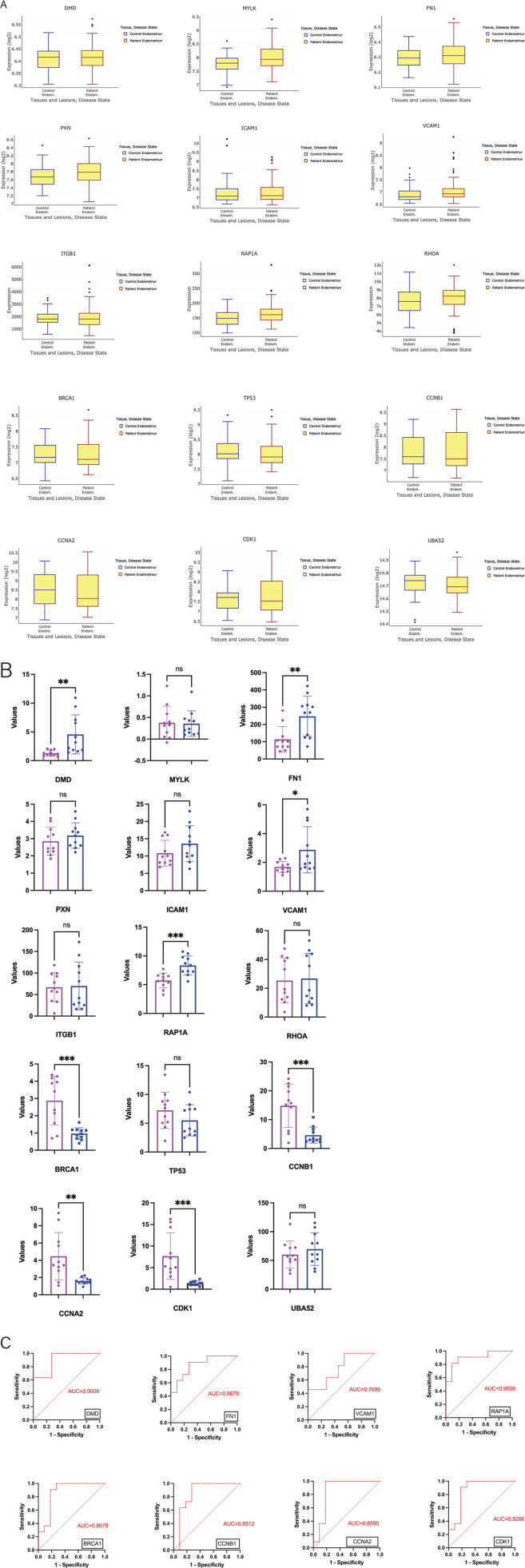
Analysis and validation of the hub m6A mRNAs in two databases. **(A)** Validation the hub m6A mRNAs in the publicly accessible ENDOMET Turku Endometriosis Database. **(B)** Validation in the GSE5108 dataset. **(C)** The ROC analysis of the eight candidate m6A mRNAs.

To further verify the findings, the authors searched the Turku Endometriosis Database to identified the expression of the 15 hub mRNAs (Figure 4A). The 15 hub mRNAs expression trends between two groups were consistent with the authors’ results, although it was not known whether the differences were statistically significant. Moreover, the GSE5108 database was applied to explore the expression level of these hub mRNAs (Figure 4B). Among the 15 hub mRNAs, eight mRNAs were highly statistically significant. Meanwhile, ROC analysis in the GSE5108 database elucidated the diagnostic significance of the eight candidate mRNAs. Their AUC values were all higher than 0.7, and these results revealed that these seven key notes maybe have certain value for diagnosing endometriosis (Figure 4C).

### Substantiation of small-molecule therapeutic drugs

**FIGURE 5:**
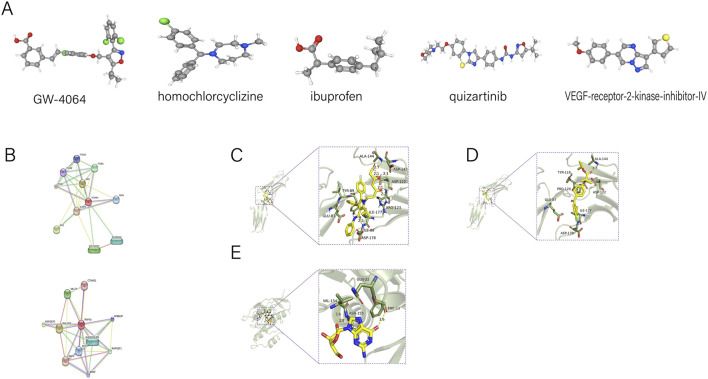
3D conformers of the candidate therapeutic agents. **(A)** The 3D structures of the five chemicals. **(B)** Chemical compounds acting on *VCAM1* and *RAP1A* in the STITCH database. **(C,D)** Docking of *VCAM1* and therapeutic. **(E)** Docking of *RAP1A* and therapeutic: Guanosine formed hydrogen bonds with *PHE-23, ASN-155 and VAL-154*.

With retrieving the CMAP database, abundant remarkable small-molecule agents targeting endometriosis were screened. The top five small-module agents with high correlations with endometriosis were GW-4064, homochlorcyclizine, ibuprofen, quizartinib, and VEGF-receptor-2-kinase-inhibitor-IV. The 3D structures of the above five chemicals that may be selected for potential treatment options were acquired from PubChem and were shown in (Figure 5A).

### Hunting the connection between candidate gene and drug

Through the analysis of eight hub mRNAs in the STITCH database, the authors revealed *VCAM1* and *RAP1A* play a vital role in endometriosis. Next step, they aimed to find chemical compounds acting on *VCAM1* and *RAP1A* to observe new treatment of endometriosis. In the compound-protein interaction network, the compound guanosine which interact with *VCAM1* and *RAP1A* were identified for subsequent analysis (Figure 5B).

### Molecular docking of the hub gene and therapeutic target

Molecular docking analysis validated atorvastatin and fenofibrate to *VCAM1* binding fractions of −5.7 kcal/mol and −5.4 kcal/mol, respectively, which indicated that there was a compelling binding effect between them. Atorvastatin interacts with *VCAM1* mainly through the formation of hydrogen bonding and hydrophobic force. Atorvastatin formed hydrogen bonds with ARG-123, ASP-122, ASP-143, ALA-144, ILE-88, and the length of hydrogen bonds were 2.2Å, 2.1Å, 2.1Å, 2.1Å, 2.7Å, 2.5Å, respectively. Atorvastatin had hydrophobic effects with ASP-178, ILE-177, TYR-89, GLU-87 (Figure 5C). Fenofibrate interacts with *VCAM1* mainly through the formation of hydrogen bonding and hydrophobic force. Fenofibrate formed hydrogen bonds with *ALA-144*, and the length of hydrogen bonds was 3.7Å. Fenofibrate had hydrophobic effects with ASP-122, TYR-119, PRO-120, ILE-177, ASP-178, GLU-87 (Figure 5D).

Molecular docking analysis validated guanosine to *RAP1A* binding fractions of −6.4 kcal/mol. Guanosine formed hydrogen bonds with PHE-23, ASN-155 and VAL-154, and the length of hydrogen bonds were 3.6Å, 2.8Å and 3.6Å, respectively. Guanosine had hydrophobic effects with *GLN-22* (Figure 5E).

### Expression of *VCAM1* and *RAP1A* in clinical tissues

**FIGURE 6:**
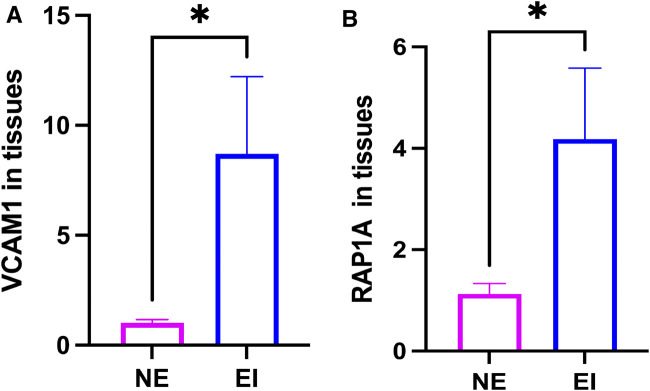
Expression of *VCAM1* and *RAP1A* in clinical tissues (12v12). **(A)** *VCAM1*. **(B)** *RAP1A*.

*VCAM1* and *RAP1A* expression in tissues were detected by qRT-PCR assay. The analysis data showed that *VCAM1* and *RAP1A* were over expressed in ectopic tissues than normal tissues (Figure 6).

## Discussion

Endometriosis has been progressing very slowly in terms of pathogenesis and early diagnosis over the years due to its symptom-driven approach diagnosis, and treatment relying on laparoscopic examination (Bulun et al., 2019). Previous studies on the pathogenesis of endometriosis have mainly focused on searching for pathogenic genes, with little research on the role of RNA in gene expression (Li et al., 2021). Similarly, due to the unclear molecular mechanism of endometriosis and limited current treatment methods, the discovery of new therapeutic targets and effective drug molecules remains a focus of research. This study used arraystar m6A-mRNA epitranscriptomic microarray, biological information analysis technologies to demonstrate the existence of abnormally expressed mRNA during the disease onset process, and screened out 15 important nodes. After being validated by other databases, eight mRNA with high diagnostic value were screened out. Based on the above results, we evaluated the correlation between molecules and genes of the drugs, and also indicated that some drugs and genes have more significant effects in treating diseases.

This study presents notable innovations. Previous studies have mainly focused on m6A regulatory proteins (writers, erasers, and readers), whereas our study directly analyzed m6A methylation at the whole-transcriptome level and performed an integrated analysis of the m6A methylome and transcriptome. We identified novel hub genes and potential targeted small-molecule compounds. Furthermore, our findings were validated at multiple levels using clinical samples and several public databases.

GO enrichment and KEGG analysis indicate that abnormal methylation affects certain functions and pathways, which may provide new bioinformatics basis for explaining the complex pathogenesis of endometriosis. The validation of m6A hypermethylated and hypomethylated genes indicates that hypermethylated genes are mainly enriched in “signaling”, “protein-containing complex binding”, “regulation of response to stimulus”, and other aspects while hypomethylated genes are mainly enriched in “cellular process” and “protein binding”. KEGG analysis results confirm that hypermethylation genes are significantly enriched in signaling pathways such as cell adhesion molecules, while the hypomethylated genes were mainly linked to cell cycle and ribosome. M6A mRNA not only directly promotes the growth and proliferation of ectopic endometrium, as well as the migration, adhesion, and release of inflammatory factors through epigenetic signaling pathways, but also plays a role in the cell adhesion pathway due to its high methylation characteristics. An experiment has observed an increase in NF-κB activity and adhesion ability in ectopic endometrium (González-Ramos et al., 2012). Compared with cultured endometrial stromal cells, the expression of intercellular adhesion molecule-1 (ICAM-1) mRNA and protein in endometriosis is enhanced (Viganò et al., 1998a). Therefore, abnormal cell adhesion is considered a key initiating factor for ectopic endometrial growth. The significant enrichment of hypermethylated m6A mRNA in pathways such as cell adhesion molecules indicates its role in the pathogenesis of endometriosis. The presence of more hypomethylation genes in ectopic endometrium indicates that hypomethylation genes play a more important role in ectopic endometrial lesions. Hypomethylation of m6A can lead to the inactivation of the AKT pathway, thereby increasing the proliferation and tumorigenicity of endometrial cancer cells (Liu et al., 2018). Therefore, the similar carcinoid nature of endometriosis and endometrial cancer allows us to reasonably infer that low methylation of m6A can have the same effect on ectopic endometrial cells. In addition, the m6A hypomethylation gene is significantly associated with the Hippo signaling pathway, and activation of this pathway can enhance cell proliferation and apoptosis (Song et al., 2016). M6A hypomethylation activated PPP2R2D and YWHAZ genes may be involved in the pathogenesis of endometriosis, while PPP2R2D and YWHAZ are involved in cell proliferation and migration (Joshi et al., 2015). Through the above two pathways, m6A hypomethylation is enriched in the cell cycle and ribosome pathway, thereby affecting endometriosis.

After protein-protein interaction network analysis and hub gene selection, a total of 15 mRNA nodes with hypermethylated-upregulated expression, hypomethylated-upregulated expression, and hypomethylated-downregulated expression were selected from them. After applying GSE5108 database and ROC analysis, eight out of 15 key nodes showed diagnostic value. Among them, FN1 is significantly associated with the risk of endometriosis through the involvement of sex steroid hormone pathways. By mediating multiple interactions between cells and extracellular matrix, its encoded fibronectin can participate in important cellular activities such as cell adhesion, migration, growth, and differentiation, and also plays a key role in cell healing, blood coagulation, and metastasis (Soikkeli et al., 2010). Multiple studies on FN1 have found that the rs1250248 SNP at the FN1 site is significantly associated with moderate to severe endometriosis (Rahmioglu et al., 2014). SICAM-1 and sVCAM-1, as glycoproteins that promote cell proliferation and neovascularization, play important roles in promoting inflammation and immune response (Vodolazkaia et al., 2012). Due to the interference of sICAM-1 with the function of natural killer (NK) cells, serum sICAM-1 may be associated with the active inflammatory stage of endometriosis (Oosterlynck et al., 1992). The differential expression and alteration of ICAM-1 in endometriosis may be involved in the mechanism of evading immune surveillance, allowing for the diffusion of endometrial cells and invading other parts of the uterus (Viganò et al., 1998b). A Study has found that sICAM-1 levels in patients with endometriosis are higher than those in unaffected patients, and the sVCAM-1/sICAM-1 ratio plays a role as a diagnostic tool for this disease (Kuessel et al., 2017). The expression imbalance of ITBG1 in patients with endometriosis affects the adhesion and invasiveness of endometrial stromal cells, leading to the onset of endometriosis. The Rho family serves as molecular switches involved in the regulation of various cellular functions, including cytoskeletal organization, growth, and differentiation. In endometriosis, the RhoA content in ectopic endometrial cells is significantly higher than that in ectopic endometrial cells, which is related to promoting the migration of endometrial cells in endometriosis (Jiang et al., 2012). At the same time, some studies suggest that the increased expression of RhoA protein in endometrial stromal cells may be involved in the pathogenesis of endometriosis related fibrosis (Yotova et al., 2011). As a tumor suppressor gene involved in DNA damage repair, BRCA1’s high-risk mutations will lead to the failure of important error free DNA repair processes (homologous directed repair), significantly increasing the risk of cancer (Moynahan and Jasin, 2010). Similar BRCA1 gene mutations have been found in multiple studies on genomic mutations in endometriosis, endometrial cancer, or ovarian cancer (Gaia-Oltean et al., 2021). The TP53 gene, as a representative tumor suppressor gene, plays an important role in regulating cell growth and preventing cancer (Kern et al., 1991). The product of the TP53 gene, the p53 protein, usually induces cell apoptosis, cell cycle arrest, or cell aging during DNA damage, thereby inhibiting the proliferation of damaged cells, maintaining genomic integrity, and preventing tumor occurrence (Robles et al., 2002). The polymorphism of the TP53 gene codon 72 is considered a risk factor for endometriosis (Song and Lee, 2014).

By using ROC analysis, we elucidated the diagnostic significance of candidate mRNA for endometriosis. By searching the CMAP database, we identified several small molecule drugs with significant therapeutic effects on endometriosis. These research results undoubtedly clarify the future research direction for the diagnosis and treatment of diseases. The survival and growth of ectopic endometrial lesions depend on the local estrogen (E2) produced by endometriotic implants, and aromatase is a key enzyme in estrogen biosynthesis. Estrogen mainly regulates its biological response through its two different estrogen receptors, ERa and ERb. ERb promotes the proliferation of endometriosis tissue and inhibits cell death by interacting with inflammatory body complexes and cytoplasmic apoptosis mechanisms, thereby promoting the progress of endometriosis (Han et al., 2015). Therefore, any method that reduces the expression and/or activity of aromatase and ERb may become a new treatment strategy for endometriosis. Farnesoid X receptor agonist GW4064 treatment inhibits the expression of aromatase and ERb by increasing the recruitment of FXR and Stat3 to the CYP19A1 and ESR2 promoter regions in ESCs (Wu et al., 2019). Ibuprofen, as a common non steroidal anti-inflammatory drug with antipyretic and analgesic properties, has been widely used for pain relief caused by endometriosis, and its therapeutic effect on the disease has also been revealed in this study. In years of research on the pathogenesis of endometriosis, the role of angiogenesis has been demonstrated. VEGF is considered a major factor in neovascularization due to its promotion of increased vascular permeability, extracellular matrix degeneration, endothelial cell migration, proliferation, and angiogenesis. Therefore, VEGF receptor-2 kinase inhibitor IV can interfere with the occurrence and development of diseases by blocking VEGF.

Molecular docking results only provide computational evidence of potential binding interactions, rather than confirmed pharmacological activity. The binding energies of VCAM1 and RAP1A were −5 to −6 kcal/mol, indicating a moderate binding affinity. Nevertheless, moderate binding may still exert biological effects within the cellular microenvironment. These compounds are regarded as preliminary candidates and require further experimental validation. Collectively, these findings offer a rational starting point and theoretical foundation for subsequent drug screening.

Through this experimental study, we have concluded that *VCAM1* and *RAP1A* are overexpressed in ectopic tissues compared to normal tissues. In previous studies, *VCAM-1* has been reported to overexpress in the serum of patients with many types of malignant tumors, such as breast cancer, gastric cancer, ovarian cancer and melanoma (Chen et al., 2011; Ding et al., 2003). In a study evaluating new biomarkers for stage III and IV endometriosis, it was found that postoperative sVCAM-1 expression decreased in patients with endometriosis, indicating that sVCAM-1 levels may be associated with the presence of ectopic endometrium (Kovalak et al., 2023). In a study on the role and mechanism of *VCAM-1* in endometriosis, it was found that *VCAM-1* mRNA levels in human endometriosis tissue were increased compared to normal endometrial tissue (Zhang et al., 2019). These studies are consistent with our conclusion that *VCAM-1* hypermethylation upregulates expression. VCAM-1/CD106 is a member of the immunoglobulin (Ig) superfamily and is believed to be an inducible cell surface glycoprotein primarily present on the endothelium. *VCAM-1* plays an important role in cellular immune response, mediating the rolling and adhesion cascade reactions of circulating white blood cells towards and across the endothelium. In addition to being expressed on the cell membrane, *VCAM-1* can also be cleaved into soluble form (sVCAM-1) from endothelial or other cell surfaces. The serum sVCAM-1 levels in patients with immune diseases and various malignant tumors increase (Osborn et al., 1989). In addition, *VCAM-1* has been reported to promote and maintain the establishment of tumor vascular systems in many types of cancer by mediating tumor cell adhesion to vascular endothelial cells and promoting metastasis processes, thereby playing a role in the progression of metastasis in these tumors (Banks et al., 1993). In this study, we found that *RAP1A* was hypomethylated and upregulated. In previous studies, *RAP1A* was overexpressed in many cancers (Bhari et al., 2021). Research has found that *RAP1A* and *EPAC1* are highly expressed in ovarian endometriosis, and their expression is positively correlated. It is speculated that *RAP1A* and *EPAC1* may cooperate and participate in the occurrence and development of EMs and the regulation of dysmenorrhea (Li WS. et al., 2022). Similarly, the conclusions we obtained in this study confirm the above conclusions. RAS related proteins (RAP) GTPases are highly expressed in platelets and are key regulatory factors for cell adhesion, cytoskeletal remodeling, and MAP kinase signaling (Ca and ron, 2003). There are five members in mammals, including RAP1A and RAP1B, and RAP1A and RAP1B are highly homologous isomers. Research has shown that RAP gtpases, especially RAP1A and RAP1B, drive platelet activation at the site of vascular injury by switching on various platelet reactions, including internal and external activation and secretion of integrins, as well as the formation of eicosane like mediators (Stefanini et al., 2012). They are key molecular switches that activate/adhere to platelets at the site of injury. RAP1A deficiency affects FGF2 related angiogenesis, reduces macrophage migration ability, bone marrow cell and lymphocyte chemotaxis, and also affects cell adhesion, migration, phagocytosis, and oxidative burst of white blood cells (Li et al., 2007). The above two types of mRNA affect the underlying pathogenesis of endometriosis through various pathways such as angiogenesis and cell adhesion, thereby participating in the growth and proliferation of ectopic endometrium. Therefore, these two types of mRNA are highly likely to be indispensable m6A related mRNA in endometriosis.

Nevertheless, two limitations of this study deserve further clarification. Firstly, the current study is constrained by the relatively limited number of clinical specimens. Subsequent research will employ expanded patient cohorts and conduct extensive functional assays together with rigorous validation to consolidate the present observations. Secondly, inconsistencies between hub genes identified via microarray profiling and those confirmed in public databases may mainly stem from discrepancies in analytical platforms and study designs. Our microarray investigation was based on a single-center cohort with a modest sample size and homogeneous clinical characteristics. By comparison, public repositories such as the GEO database often contain mixed samples with diverse ethnicities, distinct disease stages, and varied prior therapeutic interventions, which naturally results in divergent gene expression patterns and candidate hub genes. Variations in data preprocessing protocols and normalization strategies across different datasets may also account for such inconsistencies.

This study suggests that abnormal methylation of some mRNA may be involved in the pathogenesis of endometriosis. Understanding and discovering these abnormally methylated mRNA and their pathways of action will provide the possibility of discovering new biomarkers for endometriosis, and utilizing these research results may make significant contributions to discovering new treatment methods for endometriosis.

## Conclusion

In summary, with arraystar m6A-mRNA epitranscriptomic microarray, biological information analysis technologies, and validation of other databases, aberrant m6A-related mRNAs were uncovered, as well as efficient therapeutic drugs. Ultimately, we discovered that *VCAM1* and *RAP1A* may be essential m6A-related mRNA of EM. Importantly, we also searched for a few efficient small-molecule agents that provide new views for the treatment of EM. These results provide fresh insights into m6A methylation in EM and reveal potential biomarkers and precision medicine for EM.

## Data Availability

The datasets presented in this study can be found in online repositories. The names of the repository/repositories and accession number(s) can be found in the article/supplementary material.

## References

[B1] Banks R. E. Gearing A. J. Hemingway I. K. Norfolk D. R. Perren T. J. Selby P. J. (1993). Circulating intercellular adhesion molecule-1 (ICAM-1), E-selectin and vascular cell adhesion molecule-1 (VCAM-1) in human malignancies. Br. J. Cancer 68 (1), 122–124. 10.1038/bjc.1993.298 7686390 PMC1968321

[B2] Bhari V. K. Kumar D. Kumar S. Mishra R. (2021). Shelterin complex gene: prognosis and therapeutic vulnerability in cancer. Biochem. Biophys. Rep. 26, 100937. 10.1016/j.bbrep.2021.100937 33553693 PMC7859307

[B3] Bulun S. E. Yilmaz B. D. Sison C. Miyazaki K. Bernardi L. Liu S. (2019). Endometr. Endocr. Rev. 40 (4), 1048–1079. 10.1210/er.2018-00242 30994890 PMC6693056

[B4] Burney R. O. Giudice L. C. (2012). Pathogenesis and pathophysiology of endometriosis. Fertil. Steril. 98 (3), 511–519. 10.1016/j.fertnstert.2012.06.029 22819144 PMC3836682

[B5] Caron E. (2003). Cellular functions of the Rap1 GTP-binding protein: a pattern emerges. J. Cell Sci. 116 (Pt 3), 435–440. 10.1242/jcs.00238 12508104

[B6] Chen Q. Zhang X. H. Massagué J. (2011). Macrophage binding to receptor VCAM-1 transmits survival signals in breast cancer cells that invade the lungs. Cancer Cell 20 (4), 538–549. 10.1016/j.ccr.2011.08.025 22014578 PMC3293160

[B7] Ding Y. B. Chen G. Y. Xia J. G. Zang X. W. Yang H. Y. Yang L. (2003). Association of VCAM-1 overexpression with oncogenesis, tumor angiogenesis and metastasis of gastric carcinoma. World J. Gastroenterol. 9 (7), 1409–1414. 10.3748/wjg.v9.i7.1409 12854131 PMC4615473

[B8] Gaia-Oltean A. I. Pop L. A. Cojocneanu R. M. Buse M. Zimta A. A. Kubelac P. (2021). The shifting landscape of genetic alterations separating endometriosis and ovarian endometrioid carcinoma. Am. J. Cancer Res. 11 (4), 1754–1769. 33948387 PMC8085850

[B9] Giudice L. C. Kao L. C. (2004). Endometriosis. Lancet 364, 1789–1799. 10.1016/S0140-6736(04)17403-5 15541453

[B10] González-Ramos R. Defrère S. Devoto L. (2012). Nuclear factor-kappaB: a main regulator of inflammation and cell survival in endometriosis pathophysiology. Fertil. Steril. 98 (3), 520–528. 10.1016/j.fertnstert.2012.06.021 22771029

[B11] Halme J. Hammond M. G. Hulka J. F. Raj S. G. Talbert L. M. (1984). Retrograde menstruation in healthy women and in patients with endometriosis. Obstet. Gynecol. 64 (2), 151–154. 6234483

[B12] Han S. J. Jung S. Y. Wu S. P. Hawkins S. M. Park M. J. Kyo S. (2015). Estrogen receptor β modulates apoptosis complexes and the inflammasome to drive the pathogenesis of endometriosis. Cell 163 (4), 960–974. 10.1016/j.cell.2015.10.034 26544941 PMC4640214

[B13] Han X. Liu J. Cheng G. Cui S. (2020). Gene signatures and prognostic values of m6a rna methylation regulators in ovarian cancer. Cancer control. 27 (1), 7–30. 10.1177/1073274820960460 32951457 PMC7791456

[B14] Huang W. Chen T. Q. Fang K. Zeng Z. C. Ye H. Chen Y. Q. (2021). N6-methyladenosine methyltransferases: functions, regulation, and clinical potential. J. Hematology and Oncology 14 (1), 117. 10.1186/s13045-021-01129-8 34315512 PMC8313886

[B15] Jiang Q. Y. Xia J. M. Ding H. G. Fei X. W. Lin J. Wu R. J. (2012). RNAi-mediated blocking of ezrin reduces migration of ectopic endometrial cells in endometriosis. Mol. Hum. Reprod. 18 (9), 435–441. 10.1093/molehr/gas019 22544491

[B16] Jiang L. Zhang M. Wu J. Wang S. Yang X. Yi M. (2020). Exploring diagnostic m6a regulators in endometriosis. Aging 12 (24), 25916–25938. 10.18632/aging.202163 33232273 PMC7803542

[B17] Jones A. Lechner M. Fourkala E. O. Kristeleit R. Widschwendter M. (2010). Emerging promise of epigenetics and DNA methylation for the diagnosis and management of women’s cancers. Epigenomics 2 (1), 9–38. 10.2217/epi.09.47 22122746

[B18] Joshi N. R. Su R. W. Chandramouli G. V. Khoo S. K. Jeong J. W. Young S. L. (2015). Altered expression of microRNA-451 in eutopic endometrium of baboons (Papio anubis) with endometriosis. Hum. Reprod. 30 (12), 2881–2891. 10.1093/humrep/dev229 26370665 PMC4643526

[B19] Kern S. E. Kinzler K. W. Baker S. J. Nigro J. M. Rotter V. Levine A. J. (1991). Mutant p53 proteins bind DNA abnormally *in vitro* . Oncogene 6 (1), 131–136. 1846954

[B20] Kovalak E. E. Karacan T. Zengi O. Karabay Akgül Ö. Özyürek Ş. E. Güraslan H. (2023). Evaluation of new biomarkers in stage III and IV endometriosis. Gynecol. Endocrinol. 39 (1), 2217290. 10.1080/09513590.2023.2217290 37236244

[B21] Krishnamoorthy K. Decherney A. H. (2017). Genetics of endometriosis. Clin. Obstet. Gynecol. 60 (3), 531–538. 10.1097/GRF.0000000000000293 28742585

[B22] Kuessel L. Wenzl R. Proestling K. Balendran S. Pateisky P. Yotova st (2017). Soluble VCAM-1/soluble ICAM-1 ratio is a promising biomarker for diagnosing endometriosis. Hum. Reprod. 32 (4), 770–779. 10.1093/humrep/dex028 28333208

[B23] Lee Y. Choe J. Park O. H. Kim Y. K. (2020). Molecular mechanisms driving mRNA degradation by m6A modification. Trends Genetics 2020;TIG 36 (3), 177–188. 10.1016/j.tig.2019.12.007 31964509

[B24] Li Y. Yan J. De P. Chang H. C. Yamauchi A. Christopherson K. W. (2007). *RAP1A* null mice have altered myeloid cell functions suggesting distinct roles for the closely related Rap1a and 1b proteins. J. Immunol. 179 (12), 8322–8331. 10.4049/jimmunol.179.12.8322 18056377 PMC2722108

[B25] Li X. Xiong W. Long X. Dai X. Peng Y. Xu Y. (2021). Inhibition of METTL3/m6A/miR126 promotes the migration and invasion of endometrial stromal cells in endometriosis. Biol. Reproduction 105 (5), 1221–1233. 10.1093/biolre/ioab152 34382070 PMC10308507

[B26] Li H. Wang C. Lan L. Yan L. Li W. Evans I. (2022a). Mettl3 promotes oxaliplatin resistance of gastric cancer cd133+stem cells by promoting parp1 mrna stability. Cell. Mol. Life Sci. 79 (3), 135. 10.1007/s00018-022-04129-0 35179655 PMC11072755

[B27] Li W. S. Li Y. L. Cao R. Ha C. F. Sun S. Yu L. (2022b). Differential expression and bioinformatics analysis of tRF/tiRNA in endometriosis patients. Biomed. Res. Int. 2022, 9911472. 10.1155/2022/9911472 35281615 PMC8913131

[B28] Liu J. Eckert M. A. Harada B. T. Liu S. M. Lu Z. Yu K. (2018). m6A mRNA methylation regulates AKT activity to promote the proliferation and tumorigenicity of endometrial cancer. Nat. Cell Biol. 20 (9), 1074–1083. 10.1038/s41556-018-0174-4 30154548 PMC6245953

[B29] Moynahan M. E. Jasin M. (2010). Mitotic homologous recombination maintains genomic stability and suppresses tumorigenesis. Nat. Rev. Mol. Cell Biol. 11 (3), 196–207. 10.1038/nrm2851 20177395 PMC3261768

[B30] Oosterlynck D. J. Meuleman C. Waer M. Vandeputte M. Koninckx P. R. (1992). The natural killer activity of peritoneal fluid lymphocytes is decreased in women with endometriosis. Fertil. Steril. 58 (2), 290–295. 10.1016/s0015-0282(16)55224-8 1633893

[B31] Osborn L. Hession C. Tizard R. Vassallo C. Luhowskyj S. Chi-Rosso G. (1989). Direct expression cloning of vascular cell adhesion molecule 1, a cytokine-induced endothelial protein that binds to lymphocytes. Cell 59 (6), 1203–1211. 10.1016/0092-8674(89)90775-7 2688898

[B32] Rahmioglu N. Nyholt D. R. Morris A. P. Missmer S. A. Montgomery G. W. Zondervan K. T. (2014). Genetic variants underlying risk of endometriosis: insights from meta-analysis of eight genome-wide association and replication datasets. Hum. Reprod. Update 20 (5), 702–716. 10.1093/humupd/dmu015 24676469 PMC4132588

[B33] Ries R. J. Zaccara S. Klein P. Olarerin-George A. Jaffrey S. R. Pickering B. F. (2019). M6a enhances the phase separation potential of mrna. Nature 571 (7765), 424–428. 10.1038/s41586-019-1374-1 31292544 PMC6662915

[B34] Robles A. I. Linke S. P. Harris C. C. (2002). The p53 network in lung carcinogenesis. Oncogene 21 (45), 6898–6907. 10.1038/sj.onc.1205563 12362272

[B35] Soikkeli J. Podlasz P. Yin M. Nummela P. Jahkola T. Virolainen S. (2010). Metastatic outgrowth encompasses COL-I, FN1, and POSTN up-regulation and assembly to fibrillar networks regulating cell adhesion, migration, and growth. Am. J. Pathol. 177 (1), 387–403. 10.2353/ajpath.2010.090748 20489157 PMC2893681

[B36] Song G. G. Lee Y. H. (2014). A meta-analysis of the association between p53 codon 72 polymorphism and susceptibility to endometriosis. Immunol. Invest 43 (6), 595–605. 10.3109/08820139.2013.833623 24999736

[B37] Song Y. Fu J. Zhou M. Xiao L. Feng X. Chen H. (2016). Activated Hippo/Yes-Associated protein pathway promotes cell proliferation and anti-apoptosis in endometrial stromal cells of endometriosis. J. Clin. Endocrinol. Metab. 101 (4), 1552–1561. 10.1210/jc.2016-1120 26977530 PMC4880175

[B38] Stefanini L. Boulaftali Y. Ouellette T. D. Holinstat M. Désiré L. Leblond B. (2012). Rap1-Rac1 circuits potentiate platelet activation. Arterioscler. Thromb. Vasc. Biol. 32 (2), 434–441. 10.1161/ATVBAHA.111.239194 22075250 PMC3262085

[B39] Viganò P. Gaffuri B. Somigliana E. Busacca M. Di Blasio A. M. Vignali M. (1998a). Expression of intercellular adhesion molecule (ICAM)-1 mRNA and protein is enhanced in endometriosis endometrial stromal cells in culture. Mol. Hum. Reprod. 4 (12), 1150–1156. 10.1093/moehr/4.12.1150 9872366

[B40] Viganò P. Gaffuri B. Somigliana E. Busacca M. Di Blasio A. M. Vignali M. (1998b). Expression of intercellular adhesion molecule (ICAM)-1 mRNA and protein is enhanced in endometriosis *versus* endometrial stromal cells in culture. Mol. Hum. Reprod. 4 (12), 1150–1156. 10.1093/molehr/4.12.1150 9872366

[B41] Vodolazkaia A. El-Aalamat Y. Popovic D. Mihalyi A. Bossuyt X. Kyama C. M. (2012). Evaluation of a panel of 28 biomarkers for the non-invasive diagnosis of endometriosis. Hum. Reprod. 27 (9), 2698–2711. 10.1093/humrep/des234 22736326

[B42] Wang Y. Nicholes K. Shih I. M. (2020). The origin and pathogenesis of endometriosis. Annu. Rev. Pathol. 15, 71–95. 10.1146/annurev-pathmechdis-012419-032654 31479615 PMC7980953

[B43] Wu P. L. Zeng C. Zhou Y. F. Yin L. Yu X. L. Xue Q. (2019). Farnesoid X receptor agonist GW4064 inhibits Aromatase and ERβ expression in human endometriotic stromal cells. Reprod. Sci. 26 (8), 1111–1120. 10.1177/1933719118808912 30428773

[B45] Yang Y. Hsu P. J. Chen Y. S. Yang Y. G. (2018). Dynamic transcriptomic m6A decoration: writers, erasers, readers and functions in RNA metabolism. Cell Research 28 (6), 616–624. 10.1038/s41422-018-0040-8 29789545 PMC5993786

[B46] Yotova I. Y. Quan P. Leditznig N. Beer U. Wenzl R. Tschugguel W. (2011). Abnormal activation of Ras/Raf/MAPK and RhoA/ROCKII signalling pathways in eutopic endometrial stromal cells of patients with endometriosis. Hum. Reprod. 26 (4), 885–897. 10.1093/humrep/der010 21303778

[B47] Zhang J. Li H. Yi D. Lai C. Wang H. Zou W. (2019). Knockdown of vascular cell adhesion molecule 1 impedes transforming growth factor beta 1-mediated proliferation, migration, and invasion of endometriotic cyst stromal cells. Reprod. Biol. Endocrinol. 17 (1), 69. 10.1186/s12958-019-0512-9 31443713 PMC6708153

[B48] Zhao Y. Shi Y. Shen H. Xie W. (2020). m6A-binding proteins: the emerging crucial performers in epigenetics. J. Hematology and Oncology 13 (1), 35. 10.1186/s13045-020-00872-8 32276589 PMC7146974

